# Laypersons’ esthetic assessment of teeth with de- or hypomineralization – a web-based survey

**DOI:** 10.2340/aos.v84.44231

**Published:** 2025-08-19

**Authors:** Laura Ståhl, Nina Sabel, Julia Naoumova

**Affiliations:** a Department of Orthodontics, Institute of Odontology, Sahlgrenska Academy, University of Gothenburg, Gothenburg, Sweden; b Folktandvården Topas, Public Dental Service, Region Västra Götaland, Gothenburg, Sweden; c Department of Pediatric Dentistry, Institute of Odontology, Sahlgrenska Academy, University of Gothenburg, Gothenburg, Sweden; d Specialist Clinic for Orthodontics, Gothenburg, Public Dental Service, Region Västra Götaland, Gothenburg, Sweden

**Keywords:** Dental esthetics, developmental defects of enamel, dental caries, surveys and questionnaires

## Abstract

**Objective:**

To investigate young individuals’ esthetic perception of color on the buccal surface of maxillary anterior teeth, self-perception of own tooth color, and general dental esthetic estimates by using a web-based survey.

**Methods:**

A digital questionnaire was advertised on social media in 2024, targeting 18–30-year-olds in Sweden. The questionnaire included attitudes toward own tooth color, cases with molar incisor hypomineralization (MIH), fluorosis (F), white spot lesions (WSL), and general dental esthetic estimates. Chi2 assessed differences between groups, and the independent sample T-test calculated mean differences in responses. Statistical significance was set at *p* < 0.05.

**Results:**

Out of 2,082 respondents (55% women, 43% men, mean age 25.1 years), 77% rated their tooth color as acceptable, with no gender or age differences (*p* > 0.05). Pigmented fluorosis was rated as nonacceptable by 94% of the respondents, followed by cases with WSLs. At least 90% cited deviant color on a part of a tooth (DCP) as a reason for nonacceptance. Even tooth color (mean, standard deviation [SD]: 8.02, 2.36) was rated more important than white teeth (mean, SD: 7.25, 2.04), *p* < 0.001.

**Conclusion:**

De- and hypomineralization on maxillary anterior teeth are perceived negatively by young individuals in terms of dental esthetics. An even tooth color was valued higher than white teeth.

## Introduction

Tooth color has been rated by laypersons as more important than tooth shape and gingival color [1] and as more important to dental esthetics than irregular anterior teeth [2]. Adolescents and young adults consider that a good dental appearance symbolizes good health, improves attractiveness, and improves employment opportunities [3]. Furthermore, physically attractive people are assumed to have more socially desirable personalities and to be happier and more successful [4, 5]. An individual with dental decay or nonperfect dentition is often perceived by laypeople as less esthetic and less likely to be socially and professionally successful [3, 6–8]. Younger individuals are less satisfied with the color of their teeth [9], and women are less satisfied with their dental appearance and more willing than men to correct the present appearance of their teeth and perform teeth-whitening treatment [3].

Hypomineralization, a developmental defect of the enamel caused by disruption to the enamel organ during the amelogenesis process, may give rise to esthetic concerns and sometimes symptoms for the individual [10]. Examples of diagnoses of developmental defects with an impact on esthetics are molar incisor hypomineralization (MIH) and fluorosis. MIH affects the first permanent molar and frequently also the incisors. The teeth are often characterized by well-defined opacity (hypomineralization), which can vary in appearance from white to brown [11]. Fluorosis is generally seen in the dentition, that is, it is not well defined and can be mild with mottled white opacities or more severe with brown pigmentation and pitting of the enamel [12, 13]. Opacities that occur due to demineralization of the enamel by the acid environment of cariogenic bacteria are called early dental caries or white spot lesions (WSL) [14]. These are characterized by white chalky opacities at the gingival margin or buccally, where orthodontic brackets have been bonded [15]. Based on systematic reviews and a meta-analysis, the pooled prevalence of MIH is 13.5% with affected incisors seen in 36.6% of the cases [16], while the prevalence of fluorosis varies from 4.25% to 100% [17], and WSL after orthodontic treatment occurs in 27.7% to 97% of patients [18].

Children with visible enamel defects show higher rates of worry and embarrassment and perceive their own teeth as yellow and discolored [19]. White hypomineralizations have a negative impact on parents’ emotional states and attitudes, and with brown features present, they are more positive to seeking dental contact [20]. If treated, children feel more satisfied and confident and perceive their teeth as looking better [19]. Tooth color is therefore a critical factor for self-perceived satisfaction with smile appearance [21].

Oral health is defined as ‘multi-faceted and includes the ability to speak, smile, taste, touch, chew, swallow and convey a range of emotions through facial expressions with confidence and without pain, discomfort and disease of the craniofacial complex’ [22]. Additionally, oral health is a crucial aspect of overall health and well-being, both physically and mentally. It is influenced by the values and attitudes of individuals and communities [22]. Individuals with de- and hypomineralization on anterior teeth experience negative impacts on their oral health. Understanding and integrating the perspectives and attitudes of laypersons can improve the clinical management of these conditions. By considering these perspectives, crucial insights can be gained that enhance treatment outcomes and patients’ oral health.

Since no previous studies have investigated laypersons’ perception of teeth with MIH, fluorosis, and WSL the aim of the web-based survey was to investigate the esthetic perception of young individuals regarding maxillary anterior teeth with de- and hypomineralization. This included examining the base color of the teeth and the deviant color on a part of a tooth. Additionally, the survey aimed to analyze the individual’s perception of the color of their own teeth and their general dental esthetic estimates.

The null hypotheses were:

1. There is no difference from an esthetic perspective with respect to the color of teeth with different de- or hypomineralizations and teeth without opacities.
2. Having white teeth and teeth with an even color are considered equally important.
3. There is no difference between genders or between ages regarding the perception of own tooth color.

## Methods

### Study design and respondents

The study was conducted through a digital questionnaire. The inclusion criteria for participating in the study were aged between 18 and 30 years with a registered residence location in Sweden. There were no exclusion criteria; however, there were some language restrictions since the questionnaire was conducted entirely in Swedish.

### The questionnaire

A pilot of the study was pretested on both women and men (*N*: 10) of the same age as the target audience and changes were made based on their responses and comments.

First, information about the study was given, and informed consent was required from the respondents to proceed with the questionnaire (Supplementary appendix 1). The information specified that the questions were solely about the perception of tooth color and included an estimated completion time of 5–10 min. The questionnaire was completely anonymous, meaning that the authors could not see any information classified as personal data and consisted of three parts.

The first part contained questions about the respondents’ gender, age and size of their location of residence, as well as two questions concerning self-estimation of own tooth color and its evenness, to be rated on a five-point Likert scale from *Very bad* to *Very good*. There were also questions about whether the respondents had undergone any treatment for tooth color correction.

**Figure 1:**
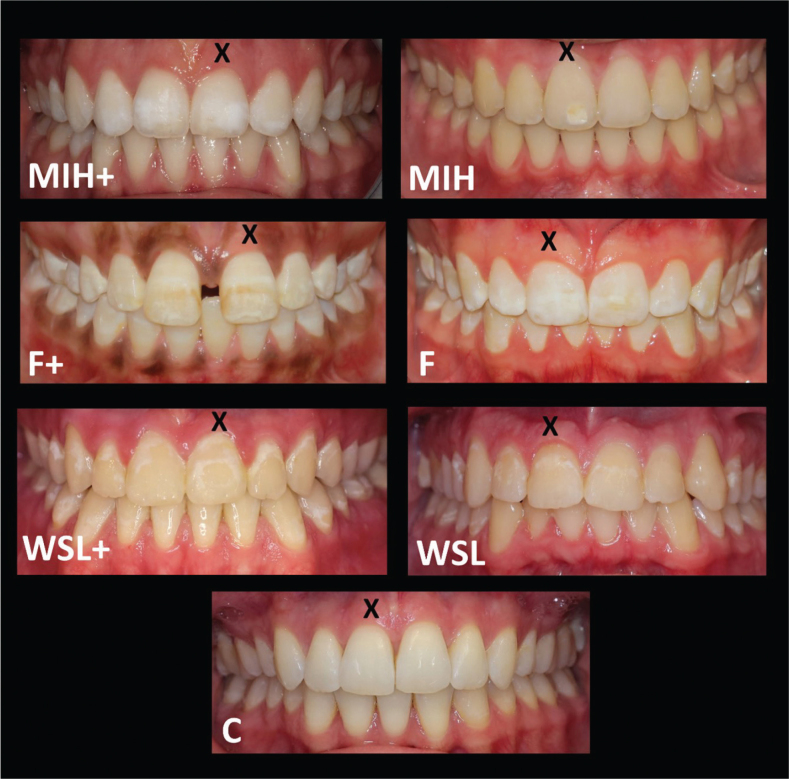
The cases in the survey with different diagnoses and severity of molar incisor hypomineralization (MIH), fluorosis (F), and white spot lesions (WSL). In the MIH and WSL cases, the ‘+’ indicates that a larger part of the surface is occupied by the de-or hypomineralization and in the F case that pigmentations are present. An additional case without hypomineralization was used as a control (C). The respondents were asked to estimate the tooth with the X mark.

The second part consisted of seven cases with intraoral front view photos representing different cases with de- or hypomineralization of the maxillary incisors and/or canines. The cases consisted of two patients each with MIH, fluorosis, and WSL and one control case without any opacity. To broaden the scope, cases with the same diagnosis had different degrees of severity (Figure 1). The two cases of MIH consisted of one with a larger area of white hypomineralization (MIH+) and one smaller with slight pigmentation (MIH). The fluorosis cases included one with brown pigmentations (F+) and another with white diffuse opacities (F). The two demineralization cases differed in size, with one presenting a larger lesion (WSL+) and the other a smaller lesion (WSL), and neither exhibited cavitation or discoloration. The respondents were asked to identify whether any tooth from 13 to 23 deviated in color. In the following question, they were asked to rate the appearance with respect to the color of a specific tooth that was marked with an X, from *Very Bad* to *Very Good* using a five-point Likert scale. Those who answered that the color was *Very good*, *Pretty good,* or *Neither good nor bad* continued to the next case. Those who had a negative perception of the tooth color received follow-up questions on whether it was the tooth base color (BC) and/or the deviant color on a part of the tooth (DCP) that was the reason for grading it as nonaccepted. Each follow-up question was answered by *Yes* or *No*. If the respondents answered yes, they were asked if the BC and DCP were too white, yellow, brown, or grey and if the opacities occupied a large part of the tooth surface.

In the last part, the respondents were asked two questions about their general estimates of tooth color. The first question was *How important do you think it is with white/light teeth?*, followed by *How important do you think it is to have teeth without a deviant color on a part of a tooth?* Answers were marked on an 11-point VAS scale from *Not important at all* (0) to *Very important* (10).

The questions were given in the same order for all respondents, and the cases were organized to avoid the same diagnosis in consecutive questions. All questions were mandatory, and it was not possible to proceed until all questions had been answered or to go back to change the answers. No compensation was paid for participation.

### Data collection

The respondents filled in the questionnaire online via the service provider Webropol AB (https://webropol.se/). It was possible for respondents to complete the questionnaire on any type of device with an available internet connection. The digital questionnaire was distributed by Meta Inc. through Meta Ads Manager, by the communications department at Sahlgrenska Academy, University of Gothenburg, Sweden, and was advertised on Facebook and Instagram to reach the target group by convenience sampling. The advertisement was first published for 4 weeks, between March 13 and April 10, 2024. As the response rate was lower among men than women, the advertisement was published during several periods to target men specifically. The advertisement headline was then reformulated to indicate that only answers from men were sought. The additional time periods were April 17 to April 24, May 13 to May 20, May 24 to June 7, and June 18 to August 31, 2024. Also, a post was made on the Academy’s profile page, where people could also respond and share the questionnaire. However, this was not the primary method for collecting responses.

### Professional assessment of DCP

A professional assessment was made by the authors regarding which teeth, from the upper right to left canines, that displayed DCP. The ImageJ software (version 1.54g, National Institutes of Health, USA) was used to calculate the proportion of DCP on the total buccal surface in per cent for the teeth marked with an X in each case by measuring the buccal surface of the tooth and the extent of DCP. Measurements were made by the authors LS, NS and JN on one occasion and 3 weeks later to assess intra- and inter-rater reliability with intra-class correlation (ICC).

### Sample size calculation

In 2023, there were 1,623,758 individuals in Sweden between 18 and 30 years of age, according to Statistics Sweden. The percentage of individuals in the different age groups varied between 7.05% and 9.19%, with 30-year-olds making up the largest group and 22-year-olds the smallest. The gender distribution was 48.08% women and 51.92% men [23]. In Sweden 2024, Meta platforms (Facebook and Instagram) were the most used social media and 41.1% of the users were in the age range of 18–34 years old. Facebook alone had 5.65 million users in total, representing 53% of the population in Sweden [24]. During the first quarter of 2024, 87% (81% of men, 93% of women) of individuals aged 16–34 years in Sweden had participated in a social networking site [25]. Therefore, social media advertisement facilitates reaching many individuals in the target group.

The method enables a large sample size, which allows for high precision. To obtain a result reflecting the perception of the target population of 18–30 years old in Sweden, with a margin of error of 2% and a confidence level of 95%, the sample group needed to be 2,398 respondents.

### Statistical analysis

In the statistical analysis, the age groups were categorized as *young adulthood* (18–25 years) and *later adulthood* (26–30 years). The size of their location of residence was divided into city (at least 200,000 inhabitants) and town (less than 200,000 inhabitants). The answers to questions, including the Likert scale to assess the respondents’ perception of their own teeth and the different cases, were grouped into *Nonacceptance* if the answer was *Very bad* or *Bad* and *Acceptance* if the answer was either *Neither good nor bad*, *Good,* or *Very good*.

The statistical analysis was performed using the Statistical Package for the Social Sciences (SPSS) software (version 28.0.1.1(15), SPSS Inc., Chicago, Illinois, USA). To analyze the categorical answers between different groups, Pearson’s chi-squared (chi^2^) test was used. To calculate the difference in the means between the replies to the questions on the general estimates, the independent sample T test was used. The statistical significance level was set at *p* < 0.05.

## Results

The advertisement for the digital questionnaire on social media reached a total of 119,382 individuals, which resulted in 5,782 clicks on the link and a start to respond 3,214 times. In total, 2,082 respondents completed the questionnaire.

All three authors were completely consistent in selecting the teeth with DCP. The ICC intra-rater value for calculating the proportion of DCP on the buccal surface of the teeth was at least 0.97 for all authors. The ICC inter-rater value for calculating the proportion of DCP on teeth between the authors was 0.99 (95% CI: 0.981–0.999).

### Respondent demographics

Table 1 shows the demographic characteristics of the respondents. Of 2,082 respondents, 55% were women and 43.3% men, with a mean age of 25.1 years. The age group with the smallest number of respondents (2.5%) was the 18-year-olds, and 24 and 28-year-olds made up the group with the highest percentage (10.7%). Most of the respondents lived in cities (50.4%).

**Table 1:** Demographic data for the respondents (N: 2,082).

Variables		*N*	%
**Gender**			
Man		902	43.3
Woman		1,145	55
Other		35	1.7
**Age (years)**			
18		52	2.5
19		61	2.9
20	*Young adulthood*	100	4.8
21		133	6.4
22		148	7.1
23		194	9.3
24		222	10.7
25		176	8.5

26		180	8.6
27	*Later adulthood*	197	9.5
28		223	10.7
29		203	9.8
30		193	9.3
**Size of location of residence**			
City (≥ 200,000 inhabitants)		1,050	50.4
Larger town (≥ 40,000 inhabitants)		656	31.5
Smaller town (< 40,000 inhabitants)		376	18.1

### Respondents’ self-perception of their teeth

**Figure 2:**
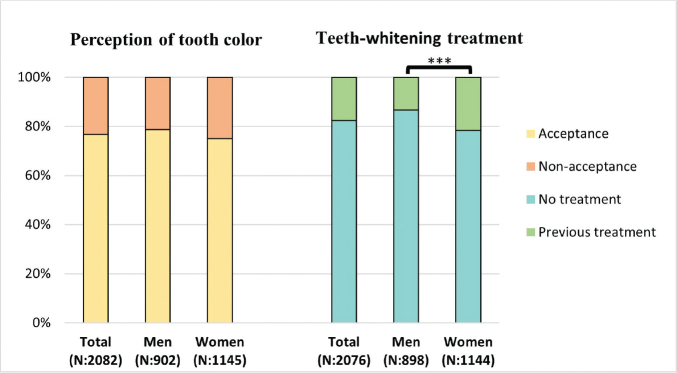
Distribution of self-perception of tooth color among the respondents and whether they have undergone previous teeth-whitening treatment. Presented as total, and men and women as percentages (%) and numbers (N). The chi2 test was calculated for acceptance/nonacceptance and for previous/no treatment, ***p < 0.001.

A large number of respondents (*N*: 1,598, 76.8%) rated their own tooth color as *acceptable.* No gender differences were seen between women (75.1%) and men (78.7%) (*p* = 0.055). However, more women (21.6%) than men (13.3%) had experience of teeth-whitening treatment (*p* < 0.001) (Figure 2). Respondents in young adulthood (76.8%) and in later adulthood (76.7%) were equally satisfied with their own tooth color (*p* = 0.962). However, the respondents in later adulthood (20.3%) had carried out teeth-whitening treatment to a larger extent than the respondents in young adulthood (15.2%), (*p* = 0.002). No difference was detected between men in young adulthood (78.8%) and later adulthood (78.7%) regarding *acceptance* of their own tooth color (*p* = 0.969) or experience of teeth-whitening treatment (15.3% and 11.3%, respectively, *p* = 0.075). Similar results were observed among women in young adulthood (74.8%) and later adulthood (75.4%) regarding *acceptance* of their own tooth color (*p* = 0.797). However, more women in later adulthood (24.4%) had carried out teeth-whitening treatment compared with women in young adulthood (18.8%), (*p* = 0.023). Six respondents were uncertain if they had undergone any whitening treatment.

A total of 36.7% of the respondents answered that they had DCP, and of them, 42.7% rated the appearance of their teeth with DCP as *nonacceptable*. More respondents with DCP (32.2%) rated their overall tooth color as *nonacceptable* compared with those without DCP (17.9%) (*p* < 0.001). No gender differences between women and men were seen, neither in the perception of having DCP (38.6% and 34.8%, respectively, *p* = 0.118), nor in *nonacceptance* of DCP (44.3% and 40.4%, respectively, *p* = 0.286). Similar results were also seen for the respondents in young adulthood and later adulthood and their perception of having DCP (37.2% and 36.2%, respectively, *p* = 0.493) and for *nonacceptance* of own teeth with DCP (43.1% and 42.4%, respectively, *p* = 0.493). Furthermore, no difference was observed between men or between women in young adulthood and later adulthood with respect to them perceiving that they have DCP (*p* = 0.232 and *p* = 0.964, respectively) or the likelihood of *nonacceptance* (*p* = 0.230 and *p* = 0.242, respectively).

**Figure 3:**
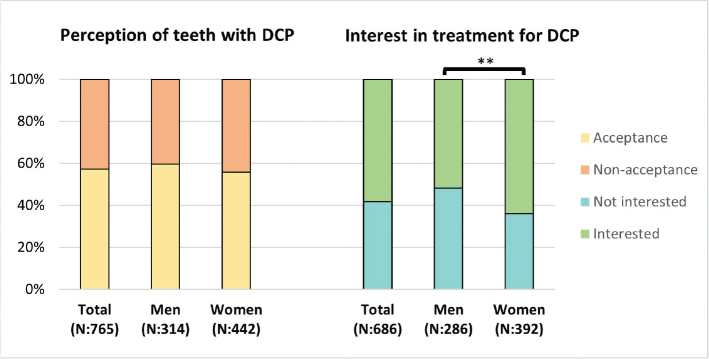
Distribution of self-perception of teeth with a deviant color on a part of a tooth (DCP). The figure also shows if the respondent would be interested in treatment for DCP. Presented as total and men and women as percentages (%) and numbers (N). The chi2 test was calculated for acceptance/nonacceptance and for interest/no interest in treatment, **p < 0.01.

Among the respondents perceiving that they have DCP, 9.2% (10.6% women, 7% men) had previously undergone treatment for DCP. Moreover, 52.2% showed an interest in the treatment of their DCP while 37.5% were not interested. A larger number (69.1%) of respondents who rated their own tooth with DCP as *nonacceptable,* compared with those who rated the tooth as *acceptable* (39.5%), were interested in getting treatment (*p* < 0.001). Women (56.6%) were more interested than men (47.1%) in having treatment for DCP (*p* = 0.002) (Figure 3). No difference could be observed between respondents in younger adulthood (50%) and later adulthood (54.6%) regarding interest in treatment (*p* = 0.11), nor was there any difference when comparing the two age groups within the genders (men, *p* = 0.440; women, *p* = 0.174). One hundred thirty respondents (6.2%) were not sure if they had DCP, and nine respondents who perceived that they had DCP were unsure whether they had or would be interested in any treatment for their DCP.

### Esthetic perception of the cases

**Figure 4:**
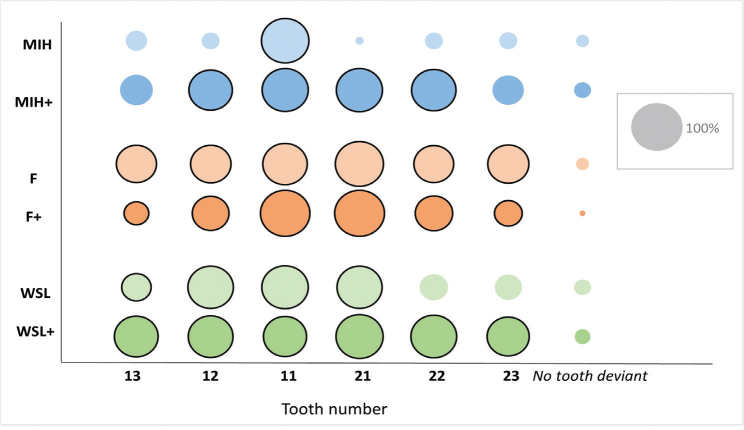
The size of the circles corresponds to the number of respondents who identified the tooth, from 13 to 23, as deviant with respect to color in the different cases of MIH (molar incisor hypomineralization), F (fluorosis), and WSL (white spot lesions). In the MIH and WSL cases, the ‘+’ indicates that a larger part of the surface is occupied by the de- or hypomineralization and in the F case that pigmentations are present. The black outline around the circles represents the teeth with deviant color on a part of a tooth (DCP). The size of the grey circle corresponds to the total number of respondents (N: 2,082).

The respondents mainly identified the teeth with DCP as deviant regarding color in all six cases. A small part of the individuals considered that no tooth was deviant in color (Figure 4).

In total, 96.5% of all respondents selected *nonacceptance* in terms of color in at least one of the cases. In addition, 40% (39.6% men and 41% women) of the respondents rated *nonacceptance* in five or more cases and a few (0.4%) in all cases (including the control case, C). There was no difference in gender (*p* = 0.501) or age (*p* = 0.117) among those who rated five or more cases as deviant.

Case F+ was rated as *nonacceptable* by most respondents (93.9 %), followed in decreasing order by WSL+, WSL, F, MIH+, MIH, and lastly C. The three cases rated as *nonacceptable* most frequently, F+, WSL+, and WSL, had the same rating order when the parameters gender, age, and size of residence location were assessed individually. In all cases (except C), respondents reported DCP more often than BC to be the only reason for the *nonacceptance* rating (*p* < 0.001). However, in the WSL and WSL+ cases, a high proportion selected both DCP and BC as reasons for *nonacceptance* (49.7% and 67.1%, respectively) (Table 2). In all cases, a reason given for *nonacceptance* of the BC was ‘too yellow’, except in case F, where the BC was assessed as being too white. Moreover, a reason given for *nonacceptance* of DCP was ‘too white’, except for case F+, where most of the respondents answered that the DCP was too brown. In all cases, except for MIH and WSL, at least 75% of the respondents answered that one of the reasons for *nonacceptance* was that the DCP occupied a large area of the tooth surface. In MIH and WSL, 67% and 50%, respectively, reported the extent of the DCP as one cause of *nonacceptance*. The largest proportion of DCP on the buccal tooth surface was in case F (89%) and the smallest in case MIH (13%) and WSL (13%) (Table 3).

**Table 2:** Descriptive statistics of the judgment of appearance with respect to color for the different cases of MIH (molar incisor hypomineralization), F (fluorosis) and WSL (white spot lesions).

*N*: 2,082	MIH+ *N* (%)	MIH *N* (%)	F+ *N* (%)	F *N* (%)	WSL+ *N* (%)	WSL *N* (%)	C *N* (%)
**Color acceptance**	993 (47.7)	1,039 (49.9)	126 (6.1)	984 (47.3)	603 (29)	924 (44.4)	2,064 (99.1)
**Color nonacceptance**	1,089 (52.3)	1,043 (50.1)	1,956 (93.9)	1,098 (52.7)	1,479 (71)	1,158 (55.6)	18 (0.9)
**Reasons for nonacceptance:**							
BC	6 (0.6)	5 (0.5)	7 (0.4)	7 (0.6)	86 (5.8)	96 (8.3)	14 (77.8)
DCP	803 (73.7)	932 (89.5)	1,611 (82.4)	841 (76.6)	392 (26.5)	468 (40.4)	1 (5.6)
Both	275 (25.3)	87 (8.3)	298 (15.2)	243 (22.1)	993 (67.1)	576 (49.7)	2 (11.1)
Other	5 (0.5)	17 (1.6)	40 (2)	7 (0.6)	8 (0.5)	18 (1.6)	1 (5.6)

In the MIH and WSL cases, the ‘+’ indicates that a larger part of the surface is occupied by the de- or hypomineralization and in the F case that pigmentations are present. F+ was rated as nonacceptable by most respondents, followed by WSL+, WSL, F, MIH+, MIH, and C. A deviant color on a part of a tooth (DCP) is presented as the main reason for nonacceptance. Very few respondents answered that either the base color (BC) or the deviant color on a part of a tooth (DCP) was the reason for nonacceptance.

**Table 3:** Descriptive statistics of the judgment of the proportion of the deviant color on a part of a tooth (DCP) for the different cases of MIH (molar incisor hypomineralization), F (fluorosis), and WSL (white spot lesions).

	Ratings by the respondents *N*: 2,082			Calculation by authors		
	Nonacceptance of color	DCP as reason for nonacceptance of color	DCP occupies a large part of the tooth as a reason for DCP nonacceptance	Proportion of DCP on the total buccal surface of the tooth		
	*N* (%)	*N* (%)	*N* (%)	Mean (%)	SD	CI (95%)
**MIH+**	1,089 (52.3)	1,078 (99)	1,046 (97)	0.413 (41)	0.011	0.401–0.425
**MIH**	1,043 (50.1)	1,021 (97.9)	681 (66.7)	0.133 (13)	0.006	0.127–0.139
**F+**	1,956 (93.9)	1,909 (97.6)	1,604 (84)	0.716 (72)	0.055	0.659–0.774
**F**	1,098 (52.7)	1,084 (98.7)	981 (90.5)	0.887 (89)	0.069	0.815–0.959
**WSL+**	1,479 (71)	1,385 (93.6)	1,041 (75.2)	0.401 (40)	0.115	0.281–0.523
**WSL**	1,158 (55.6)	1,044 (90.2)	520 (49.8)	0.126 (13)	0.017	0.108–0.149

In the MIH and WSL cases, the ‘+’ indicates that a larger part of the surface is occupied by the de- or hypomineralization and in the F case that pigmentations are present. The number (*N*) and proportion (%) of respondents who answered nonacceptance of color (left-hand column) were asked if DCP was a reason for the nonacceptance (middle column). Those who answered that the DCP was a reason were asked if the size of the DCP was a reason for the nonacceptance (right-hand column) (yes/no). The DCP proportion on the buccal surface, calculated by the authors (0.99 ICC), for the teeth marked with X in the different cases, presented with mean, SD, and 95% CI.

### General estimates

The respondents rated even tooth color (mean, standard deviation [SD]: 8.02, 2.36) as being more important than white teeth (mean, SD: 7.25, 2.04), (*p* < 0.001). Furthermore, an even tooth color was found to be more important than white teeth, regardless of gender, age, size of residence location, perception of own color (acceptance/nonacceptance), and perception of no DCP (*p* < 0.001). The same result was found among those who perceived having DCP (*p* = 0.006).

Respondents perceiving their own teeth regarding color as *acceptable* rated white teeth and an even tooth color as being *Very important* to a greater extent than those with a *nonacceptance* rating (*p* < 0.001 and *p* = 0.011, respectively). In addition, respondents without DCP rated an even tooth color as *Very important* more often than those with DCP (*p* < 0.001). In contrast, male respondents and respondents living in towns rated white teeth as being *Not important at all* more often than women or respondents living in cities (*p* = 0.034 and *p* = 0.016, respectively).

## Discussion

Since no previous studies have investigated young individuals’ perception of teeth with different forms of de- or hypomineralization, this survey was justified to contribute to the knowledge and development of oral health. The main findings indicate that teeth with de- and hypomineralization are noticed, perceived as deviant and negative for the dental appearance. Moreover, the estimates of the importance of tooth color were high, with an even tooth color being valued more than white teeth. The results from this study align well with a current topic in today’s society, esthetics. Esthetics contribute to oral health by the ability to speak, smile, and express emotions with confidence [22]. The findings emphasize the importance of being receptive to individuals’ opinions and feelings, ultimately contributing to improved oral health.

The appearance with respect to tooth color is of importance, since smiling plays an important part for how a person is perceived and how attractive they are [26, 27], as well as for self-perception and self-esteem [21]. There are several other important factors in dental esthetics, including tooth shape and position, restoration quality, and the general arrangement of the dentition [21, 28]. Tooth color has been rated of greater importance than tooth shape, gingival color, or irregular teeth [1, 2]. This is in line with the results from this study since the importance of having white teeth was rated high. Nevertheless, what was ranked as being of even greater value was having teeth with an even tooth color. Respondents who are satisfied with their tooth color were more eager to value the importance of white teeth and even tooth color high. On the other hand, those with a self-perception of DCP were more likely not to answer that it was very important with an even tooth color. The findings indicate an association between laypeople’s self-perception and the general importance of tooth color.

The age group of 18–30-year-olds that was selected to respond to the questionnaire was chosen to represent an age where dental esthetics are of great importance [9]. Furthermore, it is a group that is greatly influenced by beauty standards in society and social media [29]. In the present study, 76.8% of the respondents rated their own tooth color as *acceptable* (Figure 2). Two other studies presented similar proportions [3, 9]. However, in another study, 38.5% reported dissatisfaction with own tooth color [2]. It has previously been reported that women are more negative toward their own tooth color and less satisfied with their dental appearance than men [2, 3]. The results from this survey did not show any difference between men and women. On the other hand, more women than men would be interested in treatment for their DCP, and women had more often undergone teeth-whitening treatment (Figure 2). This might indicate an increased esthetic need among women. These findings are consistent with previous research, where women were more willing to correct and whiten their teeth than men [3]. Acceptance of one’s own dental appearance increases with age [9], and the willingness to bleach decreases [3]. This is not in line with the results from this study, probably because of the narrow age range compared with the other studies, which also investigated older individuals. Respondents in later adulthood had more often carried out teeth-whitening treatment. A possible reason for this is that teeth-whitening treatment carried out by dental professionals is not allowed below the age of 18 years in Sweden, in accordance with EU Council Directive 2011/84/EU [30]. For this reason, younger respondents would not have had the same opportunity or enough time to undergo treatment.

Almost 40% of the respondents answered that they have a DCP on at least one anterior tooth. This may be consistent with the prevalence among the population in Sweden [31, 32] because of the various types of opacities that may be present on teeth. However, it may be an overestimation as it is a layperson’s perception of a de- or hypomineralization and not a diagnosis made by a clinician. A layperson might, for example, interpret a filling as a DCP. A greater proportion of respondents with DCP compared with those without DCP rated their overall tooth color as negative and about half of them were interested in having treatment (Figure 3). This is in contrast with the findings in another study where children with MIH on incisors did not report those as having a negative effect on the esthetics [33]. On the other hand, other studies have shown that opacities on the anterior teeth seriously affect self-esteem and esthetics, and that treatment has a positive effect on patient-reported esthetics and psychosocial parameters [19, 34–37].

To broaden the scope, the same diagnosis but with different degrees of severity was included in the present survey. The young respondents identified teeth with DCP as being of deviant color to a great extent (Figure 4). This suggests that de- or hypomineralizations are noticeable feature on teeth. At least half of the respondents reported *nonacceptance* of the color in all six cases. The case with pigmented fluorosis (F+) stood out compared with the other cases, indicating that hypomineralizations with elements of brown are seen as inferior from an esthetic perspective regarding color. This agrees with previous findings suggesting that the esthetic concerns increase with increasing severity of the diagnosis [38, 39]. Although respondents were asked to judge only the color of teeth, the F+ case was the only one presented with a diastema mediale. In all other cases, the teeth were aligned. Diastema mediale negatively impacts self-perception of dental appearance [40] and might therefore had influenced the responses. Regarding WSLs, the result was consistent with other studies, suggesting that *nonacceptance* increases when the WSLs are more noticeable [8, 41]. However, in the cases with WSL, a large proportion of the respondents answered that both BC and DCP were reasons for their dissatisfaction (Table 2). This might be due to the distinct color difference between the demineralization and the base color. In the different cases, when the proportion of DCP on the buccal surface of the tooth was over 40%, a large number of the respondents noted that the DCP occupied a large part of the tooth and stated this as a reason for *nonacceptance* (Table 3), indicating that both the color and the extent of a DCP contribute to the *nonacceptance* of the color.

Cosmetic dentistry, driven by increasing consumer demand, requires careful ethical consideration to balance patient autonomy, doing good, and doing no harm while ensuring that treatments do not compromise regulatory and ethical standards [42]. The findings from this study aim to enhance understanding of the esthetic impact of de- and hypomineralizations by incorporating the views and attitudes of laypersons to further improve clinical management. However, it is important to bear in mind that asking young individuals about the perception of teeth regarding color might lead to unintended consequences such as enhanced self-criticism and dissatisfaction toward own appearance. On the other hand, young individuals live in a world influenced by social media, constantly shaped by diverse ideals of appearance.

### Limitations

Social media advertising is utilized as a recruitment strategy across various domains of healthcare research. Benefits include reduced costs, shorter recruitment periods, and improved participant selection [43–47]. Studies have also shown that participants recruited via social media are generally representative of the intended control or comparison groups [44]. One of the primary strengths of this study, which utilized a web-based approach, was the large number of respondents. However, the method inherently involves certain limitations. One drawback is the inability to select who responds. The results might therefore be influenced by laypersons with a certain interest in dental esthetics [48]. In general, men are less likely to respond to questionnaires than women [46, 49]. Another potential reason for the difficulty in reaching answers from men could be that women tend to use Facebook and Instagram more frequently [24]. Since the male response rate was low initially the advertising period was prolonged, which led to a shift regarding which individuals were targeted. In total, the questionnaire was online almost four times longer for men than for women to reach the required number of respondents. Despite that, the required sample size was not fully reached. Therefore, it might have restricted the generalizability and the precision of the findings, which should be considered when interpreting the results. However, the power calculation was based on a much greater margin of error (2%) and confidence level (95%) than usually applied.

Another limitation of the method was the selection of social media marketing. Not all individuals are active on Facebook and Instagram, and other social media might be preferable in order to reach the youngest individuals since less people in young adulthood are users of Meta platforms than in later adulthood [24]. Moreover, with social media advertising, it was not possible to go below the age of 18, nor was it possible to ensure that the questionnaire was only completed once by each individual [50]. However, because of the specific topic and scope, it is unlikely that the same person would have completed it several times. The questionnaire took considerable time to answer. This could potentially lead to stress, boredom, or loss of motivation to correctly express the opinions at the end [50, 51].

It can be difficult to glean a layperson’s true objective opinion about the appearance of teeth in terms of color. A factor that may influence the results includes the type of devices used for reviewing the images, as it was possible to complete it on any device with internet access. Both the size, screen quality and lightning of the device might play a considerable part. Additionally, respondent characteristics such as proficiency in the Swedish language, psycho-social and cognitive abilities, and color blindness can also impact the results. Another factor that might influence the results is that laypersons are not familiar with assessments of intraoral photos. An alternative would have been to use smiling images, but the disadvantage would have been that the entire tooth surface, including opacities, would not have been shown.

### Generalizability

No similar studies investigating teeth with different types of DCP objectively have previously been assessed. Even though the study did not reach its estimated sample size, the number of responses were high. Therefore, the results can still be generalized for the studied population. However, de- or hypomineralizations may be of different color, shape, and size and may not be representative of all cases. Nevertheless, the results may hint at the opinion of laypersons and the importance of an even color within dental esthetics, that is, dental caregivers should not underestimate and devalue the importance of patients’ esthetic needs.

### Further research

For future research, it would be valuable to study the responses from different age groups, both younger and older, to target the time span when DCP is noted and seen as deviant and draw conclusions on when it would be most valuable to perform treatment for DCP.

## Conclusion

This survey indicates that de- and hypomineralizations on maxillary anterior teeth are noticeable, perceived as deviant and negative for the dental esthetics. Young individuals who perceive that a part of their tooth have a deviant color are more likely to be dissatisfied with the appearance with respect to color and more interested in esthetic treatment. Moreover, the estimates of the importance of tooth color were high, where an even tooth color was valued higher than white teeth.

## Supplementary Material


