# Supplementary material for: Laypersons’ esthetic assessment of teeth with de- or hypomineralization – a web-based survey
Source: Acta Odontol Scand. 2025 Aug 19;84:44231. doi: 10.2340/aos.v84.44231 (PMC13063826; doi:10.2340/aos.v84.44231)

Supplementary material has been published as submitted. It has not been copyedited or typeset by Acta Odontologica Scandinavica.

Questions about tooth colour

English version

1. **Consent**
    - I have understood the information and give my consent to participate in the study.

Information about you

First, some questions about you. Tick the option that best suits you.

2. **What gender do you identify as?**
    - Male
    - Female
    - Other/No reply
3. **How old are you?**
    - 30 years
    - 29 years
    - 28 years
    - 27 years
    - 26 years
    - 25 years
    - 24 years
    - 23 years
    - 22 years
    - 21 years
    - 20 years
    - 19 years
    - 18 years
4. **Where do you live?**
    - Large city or municipality close to a large city (at least 200,000 inhabitants)
    - Medium-sized city or municipality close to a medium-sized city (at least 40,000 inhabitants)
    - Small town or rural municipality (fewer than 40,000 inhabitants)
5. **What is your opinion of your tooth colour in general?**

   Choose the option that best corresponds to your opinion of the colour of your teeth.
    - Very bad
    - Pretty bad
    - Neither good nor bad
    - Pretty good
    - Very good

6. **Have you undergone any treatment (at home or in a clinic) to make your tooth colour lighter/whiter?**
    - Yes
    - No
    - Don't know
7. **Do you experience that at least one of your front teeth has spots?**
    - Yes
    - No
    - Don't know
8. **What do you think about your tooth/teeth with spots?**
   Choose the option that best corresponds to what you think about the tooth/teeth. (This question was only answered if "Yes" on question 7)
    - Very bad
    - Pretty bad
    - Neither good nor bad
    - Pretty good
    - Very good
9. **Have you undergone any treatment (at home or in a clinic) for your tooth/teeth with spots?** (This question was only answered if "Yes" on question 7)
    - Yes
    - No, and not interested
    - No, but would be interested in treatment
    - Don't know

Cases

Here are some cases with questions. Choose the answer that best corresponds to your opinion.

10. **Case 1-7. Do you think that any of the highlighted teeth (marked with numbers 1-6) have a deviant colour?**
    It is possible to tick several boxes. If you think that no tooth has a deviant colour, tick the last box only.
    - 1
    - 2
    - 3
    - 4
    - 5
    - 6
    - None of the highlighted teeth has a deviant colour
11. **Case 1-7. What do you think about the colour of the tooth marked with x?**
    Look at the photo and select the option that best corresponds to your opinion of the tooth with respect to its colour.
    - Very bad
    - Pretty bad

- Neither good nor bad
- Pretty good
- Very good

12. **Case 1-7. In your answer to the previous question, you stated that the colour of the tooth marked with an X is bad. What is bad?** (This question was only answered if the previous question (11) was answered negatively; if a positive answer, the respondents moved on to the next case)
    - The base colour of the tooth (marked with an arrow)
      i. Yes
      ii. No
13. **Case 1-7. What is bad about the base colour of the tooth?**
    Choose one of the options that suits you best. (This question was only answered if "Yes" on question 12)
    - The base colour of the tooth (marked with an arrow) is to:
      i. White/light
      ii. Yellow
      iii. Brown
      iv. Grey
14. **Case 1-7. In your answer to the previous question, you stated that the colour of the tooth marked with an X is bad. What is bad?** (This question was only answered if the previous question (11) was answered negatively; if a positive answer, the respondents moved on to the next case)
    - The tooth has spots
      i. Yes
      ii. No
15. **Case 1-7. What in the spots is bad?**
    Choose one of the options that suits you best. (This question was only answered if Yes on question 14)
    - The spots are to:
      i. White/light
      ii. Yellow
      iii. Brown
      iv. Grey
    - The spot(s) cover a large area of the tooth
      i. Yes
      ii. No

General estimates about tooth colour

Estimate how important you think the colour of teeth is.

16. **How important do you think it is with white/light teeth?**
    - 0 = Not important at all
    - 1
    - 2

- 3
- 4
- 5
- 6
- 7
- 8
- 9
- 10 = Very important

17. How important do you think it is to have teeth without spots?

- 0 = Not important at all
- 1
- 2
- 3
- 4
- 5
- 6
- 7
- 8
- 9
- 10 = Very important

The Cases

Case 1

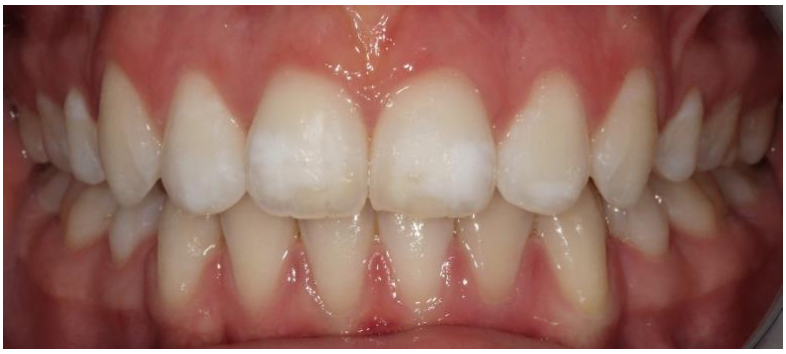

Case 2

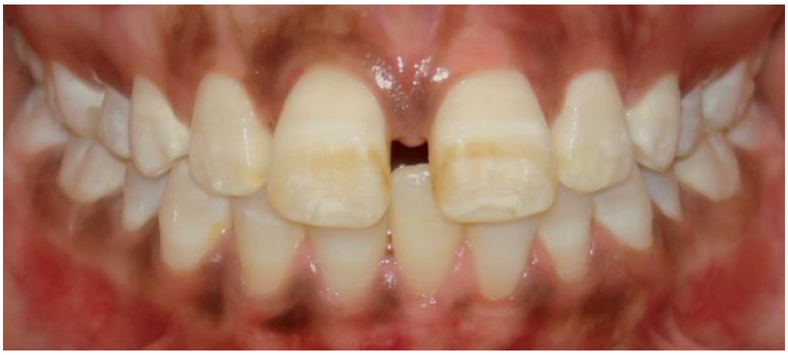

Case 3

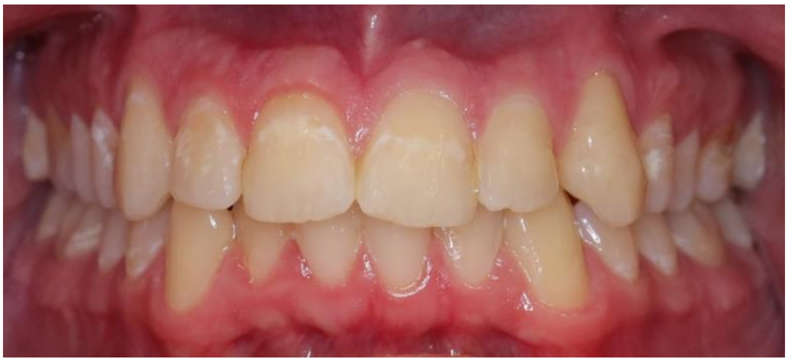

Case 4

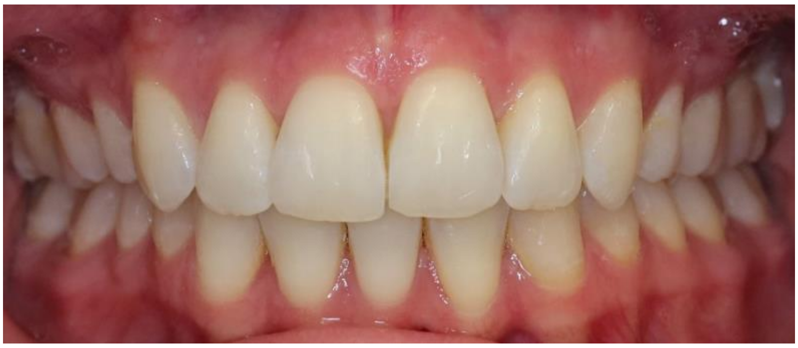

Case 5

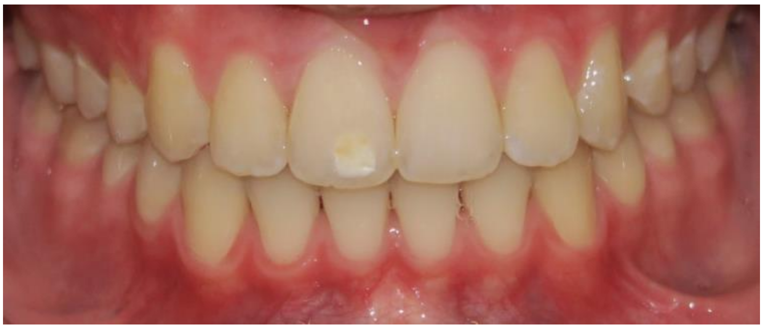

Case 6

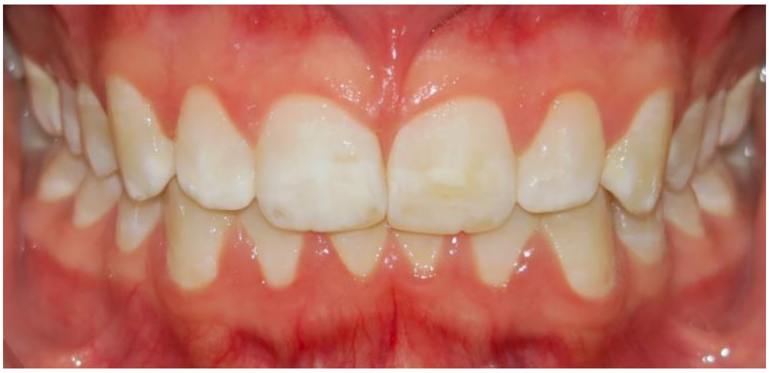

Case 7

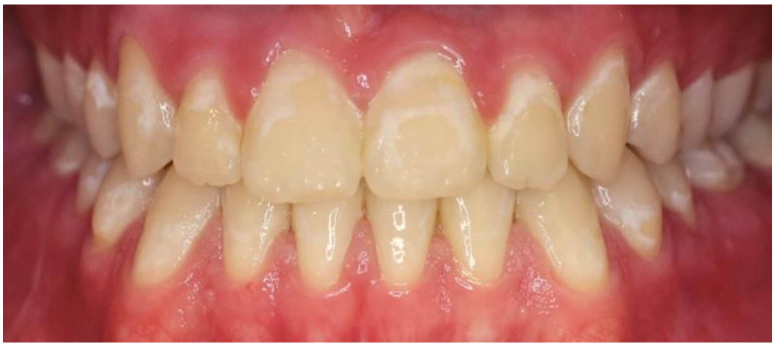

Supplement: Supplementary file 1 [file AOS-84-44231-s1.pdf]
